# Supplementary material for: The Patients’ Voice in Clostridioides difficile Infection: Large Language Model-Assisted Thematic Analysis of Patient Testimonials
Source: Open Forum Infect Dis. 2026 Oct 8;13(10):ofag650. doi: 10.1093/ofid/ofag650 (PMC13660696; doi:10.1093/ofid/ofag650)
Supplement: Supplementary file 1 [file ofag650_supplementary_data.zip › PLF_CDI_Supplemental_Figure_Table_Legends_R2.docx]

# Supplemental Figure Legends

**Supplemental Figure S1. Analytical pipeline and validation workflow.** Four-stage overview. (A) Cohort adjudication: retrieval of 204 public PLF testimonials, severity and recurrence classification, exclusion of 15 low-confidence narratives, and principal-investigator adjudication of 34 discordant cohort labels. (B) Prompt calibration and validation: 60 synthetic literature-derived cases (40 calibration across three rounds, 20 held-out validation) used to refine the coding prompt against a gold standard. (C) Dual-LLM coding run: 189 testimonials coded independently by Claude Sonnet 4.6 and DeepSeek V4 (both temperature 0), with averaged codings used for frequency and heatmap analyses. (D) Inter-rater reliability: LLM-LLM, human-LLM, and human-human comparisons, with PI adjudication of 28 discordant codings across seven testimonials.

**Alt text (Panel A):** Flowchart of cohort adjudication: 204 testimonials retrieved from the PLF testimonial library are classified by fulminant and recurrent criteria into four cohorts, recurrent fulminant n=49, fulminant non-recurrent n=18, recurrent non-fulminant n=84, and single-episode n=38, with 15 low-confidence testimonials excluded and 34 discordant cohort labels resolved by principal-investigator adjudication, yielding 189 analytic stories.

**Alt text (Panel B):** Flowchart of prompt calibration and validation: 60 synthetic literature-derived cases split into a 40-case calibration set refined over three iterative rounds and a 20-case held-out validation set, converging on a validated prompt deployed for the full corpus.

**Alt text (Panel C):** Flowchart of the dual-large-language-model coding run: the 189 analytic testimonials are coded independently by Claude Sonnet 4.6 and DeepSeek V4, both at temperature zero for deterministic output, with averaged codings used for frequency and heatmap analyses and individual model codings retained for LLM-versus-LLM concordance.

**Alt text (Panel D):** Three-circle Venn diagram of Claude, DeepSeek, and human coders alongside three inter-rater reliability comparisons: LLM-versus-LLM rated almost perfect agreement, LLM-versus-human rated moderate to substantial agreement, and human-versus-human rated substantial agreement, with 28 discordant codings across seven testimonials resolved by principal-investigator adjudication.

**Supplemental Figure S2. Acquisition source and author characteristics.** (A) Reported CDI acquisition source, full corpus (n=204): hospital-acquired 69 (34%), community-acquired 68 (33%), other or unspecified 56 (27%), unknown 11 (5%). (B) Percentage classified as fulminant by acquisition source, analytic sample: hospital-acquired 49% (n=63), community-acquired 31% (n=65), other or unspecified 25% (n=52); dashed line, overall fulminant rate (36%). Nine analytic testimonials with unknown acquisition source are not shown in Panel B (63 + 65 + 52 = 180 of 189). (C) Author type by cohort (solid bars, patient; hatched bars, caregiver).

**Alt text (Panel A):** Bar chart of CDI acquisition source across the full corpus of 204 testimonials: hospital-acquired 69, thirty-four percent; community-acquired 68, thirty-three percent; other or unspecified 56, twenty-seven percent; unknown 11, five percent.

**Alt text (Panel B):** Bar chart of the percentage of testimonials classified as fulminant by acquisition source in the analytic sample: forty-nine percent for hospital-acquired, thirty-one percent for community-acquired, and twenty-five percent for other or unspecified, against an overall fulminant rate of thirty-six percent marked by a dashed line. Nine testimonials with unknown acquisition source are not shown.

**Alt text (Panel C):** Paired bar chart of author type by cohort, solid bars for patient-authored and hatched bars for caregiver-authored testimonials. Single-episode (non-rfCDI) testimonials are entirely patient-authored (100%), while fulminant non-recurrent CDI has the highest proportion of caregiver authorship (67%).

**Supplemental Figure S3. Thematic coding prevalence by CDI cohort.** Each cell shows the percentage of testimonials within a cohort coded positive for that feature, averaged across the two independent large language model coders (Claude Sonnet 4.6 and DeepSeek V4); the same values appear numerically in Table 2. Panel A covers the eight core qualitative domains (D1–D8) and the four fulminant severity flags (F1–F4); Panel B covers the thirteen emergent semantic fields (SF01–SF13). How to read this figure: color encodes a single 0-100% scale on which near-white marks a rare feature and progressively darker red marks a progressively more prevalent one, so the darkest cells in a column are that cohort's most universal experiences; the exact percentage is printed in every cell. The four fulminant flags were codeable only in the two fulminant cohorts; the hatched gray cells marked n/a in the rCDI and non-rfCDI columns denote not applicable, not zero prevalence. Cohorts: rfCDI, recurrent fulminant (n=49); fCDI, fulminant non-recurrent (n=18); rCDI, recurrent non-fulminant (n=84); non-rfCDI, single episode (n=38).

**Alt text (Panel A):** Heatmap of eight qualitative domains and four fulminant severity flags across the four CDI cohorts, shaded from near-white at zero percent to dark red at one hundred percent, with the percentage printed in every cell. Treatment trajectory and physical symptom burden are darkest across nearly all cohorts; death or near-death is markedly darker in fulminant non-recurrent CDI than in recurrent fulminant CDI. The four fulminant flag rows are hatched gray and marked n/a for the two non-fulminant cohorts.

**Alt text (Panel B):** Heatmap of thirteen semantic fields across the four CDI cohorts, shaded from near-white at zero percent to dark red at one hundred percent. Caregiver and bereavement content is darkest in fulminant non-recurrent CDI and lightest in the other three cohorts, while bacteriotherapy shading tracks recurrent disease.

**Supplemental Figure S4. Narrative arc distribution by cohort (exploratory).** Percentage of testimonials in each cohort assigned to each narrative-arc category (Claude Sonnet 4.6 codings), one panel per cohort. The arc layer had the lowest inter-rater reliability of all coded dimensions and is reported as exploratory throughout.

**Alt text:** Four-panel bar chart of narrative-arc distribution by cohort. Recurrent fulminant CDI (n=49) is led by resolution via fecal microbiota transplantation, 16.3 percent, and near-death to recovery, 14.3 percent. Fulminant non-recurrent CDI (n=18) is dominated by caregiver burden at 55.6 percent, far exceeding any other arc in that cohort. Recurrent non-fulminant CDI (n=84) is led by resolution via fecal microbiota transplantation and self-directed recovery, each 15.5 percent. Single-episode disease (n=38) is led by adversity to resilience, 23.7 percent, and cautious hope, 18.4 percent.

# Supplemental Table Legends

**Supplemental Table S1. Full coding frequencies (%) by CDI cohort.** Per-cohort prevalence for every coded dimension (D1–D8, F1–F4, SF01–SF13), reported per model as Claude/DeepSeek with a pooled dual-model mean in the Total column. F flags are weighted by the combined fulminant cohort (n=67).

**Supplemental Table S2. Pre- versus post-calibration concordance (Coder 1 vs Coder 2, 7-item subset).** Agreement between the two human coders before and after a consensus calibration discussion, by dimension (PABAK and Gwet's AC1; delta PABAK = post minus pre). F-flag rows include only the three fulminant items.

**Supplemental Table S3. Inter-rater reliability concordance by coding dimension.** Concordance across four comparison groups (LLM-LLM, human-human, and each human coder versus the LLM reference) for binary dimensions (Panel A: D1–D8, F1–F4) and semantic fields (Panel B: SF01–SF13). Metrics are PABAK (primary), Gwet's AC1 (convergent), and Cohen's kappa (suppressed where the prevalence paradox applies).

**Supplemental Table S4. Dimensions of asymmetric inter-rater reproducibility between human and model coders.** Dimensions on which one rater type reproduced itself more consistently than the other, grouped into Cluster A (clinicians more reproducible) and Cluster B (models more reproducible), with within-type PABAK at n=16 and n=189 (model-model) and n=16 (human-human) plus a speculated mechanism.

**Supplemental Table S5. Representative patient testimonial excerpts by semantic field (SF01–SF13).** De-identified verbatim excerpts illustrating each semantic field, annotated by cohort; names, clinician identifiers, and institutions removed or generalized.
