# Supplementary material for: The Patients’ Voice in Clostridioides difficile Infection: Large Language Model-Assisted Thematic Analysis of Patient Testimonials
Source: Open Forum Infect Dis. 2026 Oct 8;13(10):ofag650. doi: 10.1093/ofid/ofag650 (PMC13660696; doi:10.1093/ofid/ofag650)
Supplement: Supplementary file 1 [file ofag650_supplementary_data.zip › PLF_CDI_Supplemental_Tables_R2.docx]

**Supplemental Table S1. Full coding frequencies (%) by CDI cohort.**

*Cohort columns show per-model coding as Claude/DeepSeek (%); the Total column is the pooled dual-model mean. F1–F4 flags were applied only to fulminant cohorts (rfCDI, fCDI); non-fulminant cohorts are not applicable (—). The total for F-flags is weighted by rfCDI + fCDI (n=67).*

| **Coding dimension** | **rfCDI** **(n=49)** | **fCDI** **(n=18)** | **rCDI** **(n=84)** | **non-rfCDI** **(n=38)** | **Total** **(n=189; n=67 for F1–F4)** |
| --- | --- | --- | --- | --- | --- |
| ***Qualitative coding domains (D1–D8)*** |  |  |  |  |  |
| D1 — Diagnostic journey | 88/86 | 78/50 | 92/76 | 100/76 | **83.7%** |
| D2 — Treatment trajectory | 100/100 | 72/67 | 100/100 | 97/97 | **96.6%** |
| D3 — Physical symptom burden | 100/100 | 94/72 | 100/93 | 100/97 | **96.6%** |
| D4 — Psychological/emotional burden | 98/98 | 94/100 | 92/92 | 74/66 | **89.5%** |
| D5 — Social/functional disruption | 78/78 | 44/39 | 60/58 | 45/58 | **60.6%** |
| D6 — Healthcare system experience | 98/98 | 78/89 | 90/89 | 74/82 | **88.9%** |
| D7 — Info-seeking / patient agency | 86/82 | 61/56 | 80/76 | 66/66 | **75.2%** |
| D8 — Recovery, resilience and outlook | 78/71 | 39/39 | 69/62 | 68/55 | **64.6%** |
| ***Fulminant severity flags (F1–F4; rfCDI and fCDI only)*** |  |  |  |  |  |
| F1 — Sepsis / organ failure | 57/57 | 67/67 | — | — | **59.7%** |
| F2 — ICU / ventilation | 14/14 | 39/33 | — | — | **20.2%** |
| F3 — Emergency surgery | 16/20 | 39/39 | — | — | **23.9%** |
| F4 — Death / near-death | 33/39 | 83/78 | — | — | **47.8%** |
| ***Semantic fields (SF01–SF13)*** |  |  |  |  |  |
| SF01 — Antibiotic trigger | 67/74 | 56/61 | 76/75 | 79/79 | **73.3%** |
| SF02 — Diagnostic failure | 51/61 | 33/39 | 44/51 | 40/40 | **47.1%** |
| SF03 — Bacteriotherapy | 61/59 | 6/6 | 46/44 | 8/5 | **37.6%** |
| SF04 — Financial / insurance burden | 31/31 | 11/11 | 32/36 | 13/13 | **26.7%** |
| SF05 — Physical severity | 100/100 | 94/100 | 77/87 | 63/76 | **85.8%** |
| SF06 — Psychological / mental health | 63/82 | 33/50 | 55/69 | 26/37 | **56.6%** |
| SF07 — Social / occupational disruption | 57/55 | 17/28 | 39/43 | 24/34 | **40.8%** |
| SF08 — Surgical / critical intervention | 26/22 | 44/56 | 4/6 | 3/5 | **14.0%** |
| SF09 — Healthcare system failure | 71/69 | 56/56 | 56/64 | 50/53 | **60.6%** |
| SF10 — Patient agency / research | 69/59 | 28/28 | 67/62 | 53/50 | **58.2%** |
| SF11 — Caregiver / bereavement | 12/12 | 67/67 | 1/4 | 0/0 | **10.6%** |
| SF12 — Long-term sequelae | 65/71 | 28/28 | 43/42 | 40/42 | **47.4%** |
| SF13 — Diet change / probiotic | 33/33 | 17/17 | 42/43 | 45/47 | **38.1%** |

*Abbreviations: rfCDI, recurrent fulminant CDI (n=49); fCDI, fulminant non-recurrent CDI (n=18); rCDI, recurrent non-fulminant CDI (n=84); non-rfCDI, non-recurrent non-fulminant CDI (n=38). D1–D8, qualitative coding domains; F1–F4, fulminant severity flags; SF01–SF13, semantic fields. —, not applicable.*

**Supplemental Table S2. Pre- vs post-calibration concordance (Coder 1 vs Coder 2, 7-item calibration subset).**

*Agreement between the two human coders on the 7-item calibration subset, before and after a consensus discussion. F-flag rows (F1–F4) include only the three fulminant-CDI items (n=3). Δ PABAK = post − pre.*

| **Coding dimension** | **n** | **Pre** **PABAK** | **Post** **PABAK** | **Δ** **PABAK** | **Pre** **AC1** | **Post** **AC1** |
| --- | --- | --- | --- | --- | --- | --- |
| ***Binary coding dimensions (D1–D8, F1–F4)*** |  |  |  |  |  |  |
| D1 — Diagnostic journey | 7 | 1.000 | 0.429 | -0.571 | 1.000 | 0.517 |
| D2 — Treatment trajectory | 7 | 0.714 | 0.143 | -0.571 | 0.835 | 0.354 |
| D3 — Physical symptom burden | 7 | 0.714 | 0.714 | +0.000 | 0.835 | 0.835 |
| D4 — Psychological/emotional burden | 7 | 0.714 | 1.000 | +0.286 | 0.835 | 1.000 |
| D5 — Social/functional disruption | 7 | 0.714 | 0.429 | -0.285 | 0.835 | 0.622 |
| D6 — Healthcare system experience | 7 | 0.429 | 0.429 | +0.000 | 0.622 | 0.622 |
| D7 — Info-seeking / patient agency | 7 | 1.000 | 1.000 | +0.000 | 1.000 | 1.000 |
| D8 — Recovery, resilience and outlook | 7 | 0.429 | 0.429 | +0.000 | 0.517 | 0.440 |
| F1 — Sepsis / organ failure | 3 | -0.333 | -0.333 | +0.000 | -0.200 | -0.200 |
| F2 — ICU / ventilation | 3 | 1.000 | 1.000 | +0.000 | 1.000 | 1.000 |
| F3 — Emergency surgery | 3 | 1.000 | 0.333 | -0.667 | 1.000 | 0.333 |
| F4 — Death / near-death | 3 | 1.000 | 1.000 | +0.000 | 1.000 | 1.000 |
| **Mean** |  | **0.698** | **0.548** | **-0.150** | **0.773** | **0.627** |
| ***Semantic fields (SF01–SF13)*** |  |  |  |  |  |  |
| SF01 — Antibiotic trigger | 7 | 0.714 | 1.000 | +0.286 | 0.736 | 1.000 |
| SF02 — Diagnostic failure | 7 | 0.429 | 0.714 | +0.285 | 0.517 | 0.736 |
| SF03 — Bacteriotherapy | 7 | 1.000 | 1.000 | +0.000 | 1.000 | 1.000 |
| SF04 — Financial / insurance burden | 7 | 1.000 | 1.000 | +0.000 | 1.000 | 1.000 |
| SF05 — Physical severity | 7 | 1.000 | 1.000 | +0.000 | 1.000 | 1.000 |
| SF06 — Psychological / mental health | 7 | 1.000 | 1.000 | +0.000 | 1.000 | 1.000 |
| SF07 — Social / occupational disruption | 7 | 1.000 | 0.429 | -0.571 | 1.000 | 0.622 |
| SF08 — Surgical / critical intervention | 7 | 1.000 | 1.000 | +0.000 | 1.000 | 1.000 |
| SF09 — Healthcare system failure | 7 | 0.429 | 1.000 | +0.571 | 0.517 | 1.000 |
| SF10 — Patient agency / research | 7 | 0.714 | 1.000 | +0.286 | 0.785 | 1.000 |
| SF11 — Caregiver / bereavement | 7 | 1.000 | 1.000 | +0.000 | 1.000 | 1.000 |
| SF12 — Long-term sequelae | 7 | 0.429 | 0.714 | +0.285 | 0.440 | 0.785 |
| SF13 — Diet change / probiotic | 7 | 0.429 | 1.000 | +0.571 | 0.440 | 1.000 |
| **Mean** |  | **0.780** | **0.912** | **+0.132** | **0.803** | **0.934** |

| PABAK > 0.80 — Almost perfect | PABAK 0.61–0.80 — Substantial | PABAK 0.41–0.60 — Moderate | PABAK 0.21–0.40 — Fair | PABAK 0.00-0.20 — Slight / Poor |
| --- | --- | --- | --- | --- |

*Abbreviations: PABAK, prevalence-adjusted bias-adjusted kappa (Byrt 1993); AC1, Gwet's first-order agreement coefficient; Δ, change (post − pre). F-flags (F1–F4) include only fulminant-CDI items (n=3).*

**Supplemental Table S3. Inter-rater reliability concordance by coding dimension. LLM x LLM: full analytic corpus (Claude Sonnet 4.6 vs DeepSeek V4; D1–D8 n=189, F1–F4 n=67 fulminant, SF n=189). Human comparisons: IRR subsample (n=16); human x human uses each coder's independent codings, and human x LLM compares each coder with the Claude Sonnet 4.6 reference codings after PI adjudication.**

**Panel A: Binary coding dimensions (D1–D8: LLM-LLM n=189, human n=16; F1–F4: LLM-LLM n=67, human n=6 fulminant items only)**

|  |  | ***LLM x LLM (Claude x DeepSeek)*** |  |  | ***Human x Human (Coder 1 x Coder 2)*** |  |  | ***Human x LLM (Coder 1 x LLM)*** |  |  | ***Human x LLM (Coder 2 x LLM)*** |  |  |
| --- | --- | --- | --- | --- | --- | --- | --- | --- | --- | --- | --- | --- | --- |
| **Dimension** | **n** | **PABAK** | **κ** | **AC1** | **PABAK** | **κ** | **AC1** | **PABAK** | **κ** | **AC1** | **PABAK** | **κ** | **AC1** |
| D1 — Diagnostic journey | 16 | **0.66** | 0.41 | 0.77 | **0.88** | 0.64 | 0.93 | **0.50** | —† | 0.68 | **0.62** | —† | 0.77 |
| D2 — Treatment trajectory | 16 | **0.99** | 0.92 | 0.99 | **0.88** | —† | 0.93 | **0.75** | —† | 0.86 | **0.88** | —† | 0.93 |
| D3 — Physical symptom burden | 16 | **0.88** | —† | 0.94 | **0.88** | —† | 0.93 | **0.88** | —† | 0.93 | **1.00** | 1.00 | 1.00 |
| D4 — Psychological/emotional burden | 16 | **0.89** | 0.72 | 0.94 | **0.62** | 0.48 | 0.71 | **0.75** | —† | 0.86 | **0.38** | —† | 0.58 |
| D5 — Social/functional disruption | 16 | **0.76** | 0.74 | 0.77 | **0.38** | 0.31 | 0.47 | **0.25** | 0.19 | 0.34 | **0.62** | —† | 0.74 |
| D6 — Healthcare system experience | 16 | **0.85** | 0.62 | 0.91 | **0.75** | —† | 0.86 | **0.62** | —† | 0.74 | **0.62** | —† | 0.77 |
| D7 — Info-seeking and patient agency | 16 | **0.87** | 0.83 | 0.90 | **0.75** | 0.59 | 0.82 | **0.50** | —† | 0.68 | **0.75** | —† | 0.84 |
| D8 — Recovery, resilience and outlook | 16 | **0.83** | 0.82 | 0.84 | **0.62** | 0.61 | 0.64 | **0.62** | 0.64 | 0.63 | **0.50** | 0.53 | 0.50 |
| F1 — Sepsis / organ failure | 6 | **0.82** | 0.81 | 0.83 | **0.33** | —† | 0.54 | **0.67** | 0.57 | 0.73 | **0.67** | —† | 0.80 |
| F2 — ICU / ventilation | 6 | **0.97** | 0.95 | 0.98 | **1.00** | 1.00 | 1.00 | **1.00** | 1.00 | 1.00 | **1.00** | 1.00 | 1.00 |
| F3 — Emergency surgery | 6 | **0.94** | 0.92 | 0.95 | **1.00** | 1.00 | 1.00 | **1.00** | 1.00 | 1.00 | **1.00** | 1.00 | 1.00 |
| F4 — Death / near-death | 6 | **0.58** | 0.58 | 0.58 | **1.00** | 1.00 | 1.00 | **1.00** | 1.00 | 1.00 | **1.00** | 1.00 | 1.00 |
| **Mean** |  | **0.838** | — | 0.866 | **0.757** | — | 0.820 | **0.712** | — | 0.788 | **0.753** | — | 0.829 |

**Panel B: Emerging semantic fields (SF01–SF13; LLM-LLM n=189, human n=16)**

|  |  | ***LLM x LLM (Claude x DeepSeek)*** |  |  | ***Human x Human (Coder 1 x Coder 2)*** |  |  | ***Human x LLM (Coder 1 x LLM)*** |  |  | ***Human x LLM (Coder 2 x LLM)*** |  |  |
| --- | --- | --- | --- | --- | --- | --- | --- | --- | --- | --- | --- | --- | --- |
| **Dimension** | **n** | **PABAK** | **κ** | **AC1** | **PABAK** | **κ** | **AC1** | **PABAK** | **κ** | **AC1** | **PABAK** | **κ** | **AC1** |
| SF01 — Antibiotic trigger | 16 | **0.93** | 0.91 | 0.94 | **0.75** | 0.74 | 0.77 | **0.75** | 0.74 | 0.77 | **1.00** | 1.00 | 1.00 |
| SF02 — Diagnostic failure | 16 | **0.83** | 0.83 | 0.83 | **0.50** | 0.51 | 0.50 | **0.25** | 0.28 | 0.26 | **0.75** | 0.74 | 0.77 |
| SF03 — Bacteriotherapy | 16 | **0.94** | 0.93 | 0.94 | **1.00** | 1.00 | 1.00 | **1.00** | 1.00 | 1.00 | **1.00** | 1.00 | 1.00 |
| SF04 — Financial / insurance burden | 16 | **0.91** | 0.88 | 0.92 | **1.00** | 1.00 | 1.00 | **1.00** | 1.00 | 1.00 | **1.00** | 1.00 | 1.00 |
| SF05 — Physical severity | 16 | **0.85** | 0.70 | 0.90 | **0.88** | 0.64 | 0.93 | **1.00** | 1.00 | 1.00 | **0.88** | 0.64 | 0.93 |
| SF06 — Psychological / mental health | 16 | **0.68** | 0.68 | 0.69 | **0.88** | 0.85 | 0.90 | **0.12** | 0.26 | 0.13 | **0.25** | 0.34 | 0.25 |
| SF07 — Social / occupational disruption | 16 | **0.79** | 0.78 | 0.80 | **0.75** | 0.71 | 0.78 | **0.25** | 0.33 | 0.26 | **0.00** | 0.20 | 0.00 |
| SF08 — Surgical / critical intervention | 16 | **0.93** | 0.85 | 0.95 | **1.00** | 1.00 | 1.00 | **0.75** | 0.67 | 0.80 | **0.75** | 0.67 | 0.80 |
| SF09 — Healthcare system failure | 16 | **0.82** | 0.81 | 0.83 | **0.50** | 0.50 | 0.51 | **0.25** | 0.25 | 0.26 | **0.75** | 0.75 | 0.75 |
| SF10 — Patient agency / research | 16 | **0.83** | 0.83 | 0.83 | **0.75** | 0.59 | 0.82 | **0.25** | 0.30 | 0.29 | **0.25** | 0.30 | 0.29 |
| SF11 — Caregiver / bereavement | 16 | **0.98** | 0.94 | 0.99 | **1.00** | 1.00 | 1.00 | **0.88** | 0.86 | 0.89 | **0.88** | 0.86 | 0.89 |
| SF12 — Long-term sequelae | 16 | **0.86** | 0.86 | 0.86 | **0.50** | 0.46 | 0.56 | **0.50** | 0.46 | 0.56 | **1.00** | 1.00 | 1.00 |
| SF13 — Diet change / probiotic | 16 | **0.94** | 0.93 | 0.94 | **0.75** | 0.71 | 0.78 | **0.75** | 0.71 | 0.78 | **1.00** | 1.00 | 1.00 |
| **Mean** |  | **0.867** | — | 0.879 | **0.788** | — | 0.811 | **0.596** | — | 0.616 | **0.731** | — | 0.744 |

| PABAK > 0.80 — Almost perfect | PABAK 0.61-0.80 — Substantial | PABAK 0.41–0.60 — Moderate |
| --- | --- | --- |
| PABAK 0.21–0.40 — Fair | PABAK 0.00-0.20 — Slight / Poor |  |

**PABAK:** Prevalence-Adjusted Bias-Adjusted Kappa (Byrt 1993). Primary metric: robust to prevalence paradox. Landis & Koch thresholds applied.

**AC1:** Gwet's AC1 (Gwet 2008). Alternative chance-correction robust to prevalence paradox; reported for convergent validity.

**κ:** Cohen's kappa. Where |kappa - PABAK| > 0.30 (prevalence paradox), kappa is shown as —† and should not be interpreted; PABAK and AC1 are preferred.

**H x H:** Human x Human (Coder 1 x Coder 2): primary inter-rater reliability estimate.

**LLM x LLM:** Claude Sonnet 4.6 vs DeepSeek V4 (DeepSeek-Chat); full analytic corpus (D1–D8 n=189, F1–F4 n=67 fulminant only, SF n=189). Both models run at temperature 0; codings are independent (no cross-model feedback).

**H x LLM:** Human x LLM: alignment of each human coder independently with the LLM reference coder (Claude Sonnet 4.6).

**Arc: Narrative-arc agreement is exploratory;** percent agreement: Coder 1 x Coder 2 68.8%, Coder 1 x LLM 37.5%, Coder 2 x LLM 43.8%.

**Supplemental Table S4. Dimensions of asymmetric inter-rater reproducibility between human and model coders. Values are PABAK unless marked; model vs model is raw Claude Sonnet 4.6 against raw DeepSeek V4, human vs human is coder 1 vs. 2.**

**Cluster A. Human raters are more reproducible than the models**

*On these dimensions, the two clinicians agreed with each other more than the two models did, which may reflect clinical training applied to content that requires interpretation beyond the explicit text.*

| **Dimension** | **Model vs model (n=16)** | **Model vs model (n=189)** | **Human vs human (n=16)** | **Speculated mechanism** |
| --- | --- | --- | --- | --- |
| SF07 Social / occupational disruption | 0.25 | 0.79 | **0.75** | Both coders reliably read indirect cues, such as an inability to work or being housebound, as disruptions. The models recovered this implicit signal inconsistently, agreeing with each other only weakly on the hard items. |
| SF06 Psychological / mental health | 0.50 | 0.68 | **0.88** | Clinicians appear to infer psychological burden from tone and described disruption and agree on it, while the models required explicit mental-health language and varied when it was absent. |
| D1 Diagnostic journey | 0.63 | 0.66 | **0.88** | Both coders are senior internal medicine residents, for whom interpreting diagnostic and therapeutic journeys, including their complexities and pitfalls, is routine clinical work. This shared expertise may support a consistent reading of the diagnostic-journey narrative that the models reproduced only moderately at either sample size. |
| SF10 Patient agency / research | 0.63 | 0.83 | **0.75** | A weaker form of the same pattern: the coders recognized self-advocacy in the described actions somewhat more consistently than the models did. |
| Narrative arc † | 50.0% | — | **68.8%** | The coders converged on a single arc more often than the models did, consistent with a shared reading of the trajectory. Exploratory. |

**Cluster B. Models are more reproducible than human raters**

*On these dimensions, the two models, run deterministically at temperature 0, reproduced one another more consistently than the clinicians, who varied in where they set a threshold or which single frame they selected.*

| **Dimension** | **Model vs model (n=16)** | **Model vs model (n=189)** | **Human vs human (n=16)** | **Speculated mechanism** |
| --- | --- | --- | --- | --- |
| SF12 Long-term sequelae | **1.00** | 0.86 | 0.50 | Humans differed on what counts as long-term, ranging from lingering symptoms to explicit chronicity. The models applied a consistent threshold and agreed completely on the edge items. |
| SF02 Diagnostic failure | **0.75** | 0.83 | 0.50 | Humans split on the threshold for diagnostic failure, with one coder counting any reported delay. The models applied it consistently. |
| SF09 Healthcare system failure | **0.75** | 0.82 | 0.50 | Humans differed on whether system failure requires a structural failure or includes an individual clinician error; the models were consistent. |
| D4 Psychological / emotional burden | **0.88** | 0.89 | 0.63 | The broad emotional-burden domain was coded reproducibly by the models but variably by humans. The narrow field SF06 shows the opposite, so the effect appears domain-specific. |
| D8 Recovery, resilience and outlook | **0.88** | 0.83 | 0.63 | A whole-narrative judgment of recovery drifted between human readers but was reproduced by the deterministic models. |
| SF13 Diet change / probiotic | **1.00** | 0.94 | 0.75 | The models were fully reproducible; the coders were substantial but not perfect, differing on borderline dietary and probiotic mentions. |
| Dominant dimension † | **62.5%** | — | 37.5% | Selecting one dominant frame was reproduced by the models far more often than by the coders, and is the basis of Figure 3. The coders agreed with the model more often than with each other. |

**Note.** D5 Social/functional disruption is not listed in either cluster. Agreement was low for both rater types on the edge items (models: 0.50; humans: 0.38), and the larger full-corpus model value (0.76) does not hold for the harder cases. D5 is thus a target for instrument refinement.

(1) PABAK, prevalence-adjusted bias-adjusted kappa (Byrt 1993).

(2) The n=16 set is the inter-rater reliability subsample selected to represent coding difficulty; n=189 is the full analytic corpus. Edge items are harder, so full-corpus model agreement runs higher. D5 illustrates this difference (0.76 corpus, 0.50 edge).

(3) † Arc and dominant dimension are multi-class and reported as percent exact agreement, not PABAK.

(4) The fulminant flags F1 to F4 rest on ten or fewer items at n=16 and are not interpreted here.

**Supplemental Table S5. Representative patient testimonial excerpts by semantic field (SF01–SF13).**

*Excerpts are drawn from PI-reviewed testimonials; names, clinician identifiers, and institution names have been removed or generalized. Cohort abbreviations: rfCDI = recurrent fulminant CDI; fCDI = fulminant non-recurrent CDI; rCDI = recurrent non-fulminant CDI.*

**SF01 — Antibiotic trigger**

| **Excerpt** | **Cohort** |
| --- | --- |
| *“I was prescribed several rounds of antibiotics by health care professionals for what they diagnosed as SIBO (whether I actually had SIBO or not, I still don’t know). Due to those antibiotics, I was dealt one of the worst cases of C. diff doctors in the Boston area had ever seen in someone my age. Six months and four rounds of the strongest antibiotics around to try and stop the infection to no avail.”* | rfCDI |
| *“After a visit to my Primary Care Provider (PCP) for a possible sinus infection, I was prescribed antibiotics and referred to my dentist for a possible tooth problem. Two weeks later, I saw my dentist who did some dental work and prescribed clindamycin. No sooner than I completed the antibiotics, I woke up in the middle of a Friday night with chills, fever, vomiting, and uncontrollable diarrhea.”* | rCDI |

**SF02 — Diagnostic failure**

| **Excerpt** | **Cohort** |
| --- | --- |
| *“After several hours, I finally saw a doctor, who ordered an ultrasound to […] rule out that what I was experiencing was not due to an ectopic pregnancy or a miscarriage. They did not run any other tests, despite my asking, as they were just too busy with their one other patient in the ER that night. I continued to decline physically, and after several rounds of fluids [..], I was sent home with a general diagnosis of a stomach bug, saying it will clear up on it’s own.”* | fCDI |
| *“It took until June 30, 2025 to be diagnosed because the gastroenterologist I saw twice insisted there was absolutely nothing wrong with me, refusing to run any tests because it would be “a waste of time.” My present gastroenterologist was the one to diagnose me and is now treating me.”* | rCDI |

**SF03 — Bacteriotherapy**

| **Excerpt** | **Cohort** |
| --- | --- |
| *“They researched my history and sent a full review of what they felt was best for me, and they recommended a fecal microbiota transplant (FMT). So on my fifth stay in the hospital, the doctors read the recommendation and put me in contact with the only gastrointestinal doctor here in Las Vegas that performs FMTs.”* | rfCDI |

**SF04 — Financial / insurance burden**

| **Excerpt** | **Cohort** |
| --- | --- |
| *“I have no savings, no career and am currently fighting for approval of permanent disability. I’ve lost my health insurance and am now on Medicaid, living in my childhood home that my family owns and lets me live in. This disease is so brutal that without my family and Boyfriend to save me, C. diff would have also made me homeless.”* | rfCDI |
| *“I was able to get insurance through the Affordable Care Act beginning on January 1, 2014. However, this has its restrictions; if I earn more than $150 in any single month, it compromises my eligibility for the insurance. I have spent all of my personal resources (money that I had saved for law school and my daughter’s wedding), and I now face uncertain financial times because I was also recently denied disability.”* | rCDI |

**SF05 — Physical severity**

| **Excerpt** | **Cohort** |
| --- | --- |
| *“Upon leaving, I was prescribed Clindamycin (a strong antibiotic) in the event I develop an infection. Mild pain and diarrhea soon began to interrupt my daily routine, which quickly escalated into debilitating pain, severe dehydration, and uncontrollable diarrhea. I was hospitalized and diagnosed with C. diff, which I had never heard of before.”* | rfCDI |
| *“I began having diarrhea that evening, and it progressed throughout the night and the next day, Sunday. By Sunday night, the 28th, I was on the floor of our living room with debilitating stomach pain after a recent trip to the bathroom. I was taken to the Emergency Room […], I had severe pain and diarrhea at that point, and was also extremely dehydrated.”* | fCDI |

**SF06 — Psychological / mental health**

| **Excerpt** | **Cohort** |
| --- | --- |
| *“But what most people don’t talk about is the emotional side of C. diff. The anxiety, panic over every gurgle or bathroom trip, and the isolation that comes from being scared to leave home have been incredibly hard to cope with. I’m currently in therapy because I’ve realized I’ve developed a fear of going out, something I never struggled with before.”* | fCDI |
| *“This is one of the reasons I left my job and decided to work from home. You are never fully warned about the long-term damage that antibiotics can cause to the gut flora or the traumatic experience of C. difficile. What they don’t tell you about is the emotional trauma (it’s true, we all stress we are going to have a relapse), the financial loss or the stress of managing a job whilst being severely ill, and the long term lasting effects that you have to deal with after.”* | rCDI |

**SF07 — Social / occupational disruption**

| **Excerpt** | **Cohort** |
| --- | --- |
| *“It also had negative effects on my relationships with my family and friends. I planned to go back to work after three months but had to cancel all of my clients because I was suffering with a recurrent infection. When I could finally return after six months I had lost a lot of my client base that I had spent so long building.”* | rfCDI |
| *“I couldn’t go to class or work and I barely left my bed — only to use the bathroom. I was enrolled full-time at a University and working two jobs and I could barely push through due to the pain I was experiencing. I felt like my friends, coworkers, professors, and family members could not understand what was going on, and when I explained I just felt embarrassed and humiliated by my awful side effects.”* | rCDI |

**SF08 — Surgical / critical intervention**

| **Excerpt** | **Cohort** |
| --- | --- |
| *“I spent three weeks in a drug-induced coma while on a ventilator. I had four surgeries while I was in the ICU; the first was a removal of my entire colon, in which I was given an ileostomy. The second surgery was to open up my abdomen in an attempt to reduce the pressure on my organs, which were failing due to the effects of severe sepsis and abdominal compartment syndrome.”* | rfCDI |

**SF09 — Healthcare system failure**

| **Excerpt** | **Cohort** |
| --- | --- |
| *“I knew that I must have been dehydrated, and at some point I was no longer able to urinate, despite having ingested plenty of liquids. I went to the emergency room, and was finally seen after being ignored in the waiting room for 45 minutes. I was admitted by the triage nurse “stat with emergent code blue.” She couldn’t even get a blood pressure reading from me.”* | rfCDI |
| *“My battle with C. diff started when I cut my ankle and was prescribed multiple antibiotics to prevent infection. About a week after finishing my last antibiotic, I began experiencing symptoms of C. diff. I visited a gastroenterologist, and while waiting for test results, she advised me to take Immodium, which I later discovered was the most detrimental course of action.”* | rCDI |

**SF10 — Patient agency / research**

| **Excerpt** | **Cohort** |
| --- | --- |
| *“When my journey began, I was completely unaware of C. diff. Now, I have an understanding of the disease, became my own advocate and through my own research, learned effective strategies to combat it. While my story may not be as extraordinary as others I’ve read about, I firmly believe that my proactive approach and self-advocacy played a pivotal role in achieving what has so far been a favorable outcome.”* | rCDI |

**SF11 — Caregiver / bereavement**

| **Excerpt** | **Cohort** |
| --- | --- |
| *“The very woman I made sure I spent time with and planned to spend more time with after I retired was sick, but I didn’t know how serious it was until I spent days and hours with her in the hospital alternating shifts with my father from 9am – 9am. My father loved my mother so much he wouldn’t leave her side or take a break. My mother would have to force him to go eat because she worried about him.”* | rfCDI |
| *“I am writing this story for my Mom […] My mom was 64 years young when she died from C. diff colitis on August 5, 2012. I was 27 years old at the time. My mom had long suffered from chronic obstructive pulmonary disease (COPD) and bouts of pneumonia since my father passed away of cancer in 2009.”* | fCDI |
| *“She had an Ileostomy done in March 2009. It saved her life, but she was never the same mentally or physically. For seven years, she suffered with multiple illnesses and, ultimately, passed away on November 16, 2016.”* | rCDI |

**SF12 — Long-term sequelae**

| **Excerpt** | **Cohort** |
| --- | --- |
| *“Now, 2024, it’s been 5 years since my 2nd transplant. 5 years later I am no longer hoping or praying for healing. I know that isn’t coming now. Now, I just pray that I will be approved for permanent disability so I am no longer a financial burden to my family and Boyfriend.”* | rfCDI |
| *“It was the worst illness I’ve ever had and to this day, 17 years later, I’m still deathly afraid of taking any medication or being around sick people. I learned my lesson but still, it’s scary how so many people don’t wash their hands and they can transmit this deathly disease to others. I have to take a probiotic (Culturelle) every day or else my stomach is upset.”* | rCDI |

**SF13 — Diet change / probiotic**

| **Excerpt** | **Cohort** |
| --- | --- |
| *“My sister pleaded to have her discharged with an adequate supply of vancomycin to allow for a slow titration down from treatment. Although the rehab facility had been administering lactobacillus twice daily, I learned only Lactobacillus GG and Saccharomyces boulardii were two probiotics that showed promise. I went out on FMLA and returned to New York for three weeks to help my sister get mom home and ensure a consistent diet of gut rebuilding foods, including prebiotics.”* | rfCDI |
| *“I was not going to get sick again, and I was willing to commit to healing by whatever method possible. After the 8 week period, I slowly reintroduced more foods, including building my gut bacteria back up with all kinds of fermentation strains: sourdough, sauerkraut, water kefir, 2 types of kombucha, etc. anything to get an abundance of probiotics in my body. Another thing I did was make sure to cut out any and all meat products from our diet that was not antibiotic-free.”* | fCDI |
